# INVESTIGATION INTO THE PREDICTORS OF HELP-SEEKING BEHAVIOR IN OLDER ADULTS

**DOI:** 10.1093/geroni/igac059.2015

**Published:** 2022-12-20

**Authors:** Michael Kann, Silvia Chapman, Martina Azar, Jillian Joyce, Leah Waltrip, Shaina Shagalow, Sandra Rizer, Stephanie Cosentino

**Affiliations:** Columbia University, Pittsburgh, Pennsylvania, United States; Columbia University Irving Medical Center, New York City, New York, United States; VA Boston Healthcare System, Boston, Massachusetts, United States; Columbia University, New York City, New York, United States; Columbia University Medical Center, New York, New York, United States; Columbia University, New York City, New York, United States; Columbia University Medical Center, New York, New York, United States; Columbia University Medical Center, New York, New York, United States

## Abstract

Early help seeking (HS) among patients with emerging Alzheimer’s disease and related dementias (AD/ADRD) can have considerable implications for treatment course, access to clinical trials, lifestyle, and future quality of life. Previous studies in older adults suggest cognitive impairment itself does not lead people to seek help; rather, HS appears to be driven by Subjective Cognitive Decline (SCD), personality, and mood. It is possible, however, that tests used to measure objective cognition were not sensitive to detect subtle cognitive impairments that may influence HS behavior. In this study of 142 cognitively healthy older adults, we examined if utilizing cognitive tasks sensitive to preclinical AD (i.e., short term memory binding, associative memory, and susceptibility to semantic interference) revealed an independent association between objective cognition and HS, or if SCD continued to be a primary driver. Participants were assessed for HS, SCD severity, personality traits (conscientiousness and neuroticism), depressive symptoms, and demographics (age, gender, education). Partially and fully adjusted regression models were conducted to examine the association between cognitive tests and HS whilst adjusting for demographics, personality, depressive symptoms, and SCD. Associative memory was the only cognitive marker significantly worse in those who help seek (B=- 0.07, SE=0.03, p=.031); however, it did not withstand adjustment for SCD. Only increased SCD (B=0.06, SE=0.02, p=.005) and educational attainment (B=0.42, SE=0.15, p=.005) had independent associations with HS. Ongoing work is establishing possible moderators of these associations to unravel the multifaceted influences on HS and to guide strategies to increase HS in older adults with SCD.

